# Germline specification from pluripotent stem cells

**DOI:** 10.1186/s13287-022-02750-1

**Published:** 2022-02-21

**Authors:** Chunmeng Yao, Ruqiang Yao, Haining Luo, Ling Shuai

**Affiliations:** 1 Tianjin Key Laboratory of Human Development and Reproductive Regulation, Department of Gynecology, Tianjin Central Gynecology and Obstetrics Hospital, Nankai University, Tianjin, 300199 China; 2 State Key Laboratory of Medicinal Chemical Biology and College of Pharmacy, Nankai University, Tianjin, 300350 China; 3 National Clinical Research Center for Obstetrics and Gynecology, Peking University Third Hospital, Beijing, 100191 China

**Keywords:** Germline, Primordial germ cells, PGC specification, Signaling pathways, Pluripotent stem cells

## Abstract

Reproduction is a key event in life guaranteeing the propagation and evolution of a species. Infertility caused by abnormal germ cell development is a topic of extensive concern. Herein, in vitro germline specification studies provide a modeling platform to investigate gametogenesis. The differentiation of pluripotent stem cells (PSCs) into germ cells has been studied for more than 30 years, and there have been many astonishing breakthroughs in the last decade. Fertile sperm and oocytes can be obtained from mouse embryonic stem cells (ESCs) through a primordial germ cell (PGC)-based method. Moreover, human PGC-like cells (PGCLCs) can be derived with a similar strategy as that used for mouse PGCLC derivation. In this review, we describe the reconstitution of PGCs and the subsequent meiosis, as well as the signaling pathways and factors involved in these processes.

## Introduction

In mammals, gametes, as the origin cells of a new organism, carry parental genetic and epigenetic information and ensure that parental information can be passed down across generations. Primordial germ cells (PGCs) are the precursor cell type of functional gametes, which are the first germline cell population in many mammals and specialize in early embryo development [1]. They migrate to the gonads and further specialize to either sperm or oocytes [2]. However, PGC specification from pluripotent stem cells (PSCs) is complicated and elusive,thus, the study of PGC reconstitution and subsequent meiosis in vitro is very important for the investigation of germline development. In addition, there are many patients without functional sperm or eggs who desire to have healthy genetically related offspring, and studies in this area are therefore in strong clinical demand. Attempts to produce gametes in vitro began in the early 2000s. Embryonic stem cells (ESCs) are PSCs with self-renewal ability and the potential to differentiate into diverse lineages [3, 4]. Whether ESCs could yield germline lineages and even functional gametes in vitro has raised broad concerns. Recently, mouse PGC-like cells (PGCLCs) were obtained from ESCs or induced pluripotent stem cells (iPSCs) in vitro through “Epiblast-like cells (EpiLCs) aggregate”-based induction and selection for specific markers [5, 6]. These derived PGCLCs can further differentiate into functional sperm or oocytes when transferred to the gonads in vivo or directly induced with specific chemical compounds and growth factors in vitro [7–9]. These were exciting breakthroughs for basic studies of development and reproductive medicine. In addition, more genetic and epigenetic information on human germlines has been revealed through the development of single-cell sequencing technologies [10, 11], greatly facilitating studies of PGC specification in humans. Here, we review the specification of the germline in recent decades in detail.

## Development of germline in mice and human

The mouse PGCs (mPGCs) arise from a portion of the post-implantation epiblast [12], responding to certain specific factors. PGCs require epiblast cells to enter the germ cell fate rather than the somatic cell fate. In mice, around embryonic day 5.25–6.25 (E5.25–6.25), bone morphogenetic protein 4 (BMP4) from the adjacent extraembryonic ectoderm began to induce the production of PGC precursors in the proximal epiblast cell population [13] instead of changing into somatic cells. In the extraembryonic mesoderm of embryos from E7.0–E7.25, BMP4-triggered cells expressing *Blimp1* (also known as *Prdm1*) [14], *Tfap2C* (also known as *AP2γ*) [15], *Prdm14* [16] and *Stella* [17] can be detected by alkaline phosphatase (AP) staining. These AP-positive cells in this stage are named PGCs. PGCs expand and appear as clusters on the amniotic membrane at approximately E7.25 [18] and then migrate to the genital ridge beginning on E9, where they further develop to the next stage [19]. Male germ cells enter meiosis from mitosis beginning at E13.5, whereas female germ cells arrest at an early stage of meiosis until adult age to resume meiosis [2].

Although the development of PGCs in mice is well known, the study of human PGCs (hPGCs) is still rare due to ethical difficulties in obtaining samples from early human embryos. Comparable to E6.25 to E7 stage embryos in mice, 2-week human embryos contain an epiblast and hypoblast, and the cytotrophoblast surround the epiblast. The amnioblast from the epiblast forms the amnion, containing the cytotrophoblast to form amniotic cavity [20]. With the beginning of gastrulation at 3 weeks, hPGCs have already formed. At approximately 4 weeks to 6 weeks, hPGCs start to migrate to the gonad gradually. Some genes involved in regulating hPGC formation have been studied in vitro to avoid ethical issues. Conserved genes such as *Blimp1*, *Tfap2C*, *Nanos3*, *DDX4*, *Dazl* and *Nanog* are highly expressed in both mice and humans. However, *Sox17* is specifically expressed in hPGCs rather than in mPGCs [21]. Therefore, it is necessary to address the key modules and pathways involved in the generation of hPGCs.

## Differentiation of germline from ESCs

**Fig. 1.**
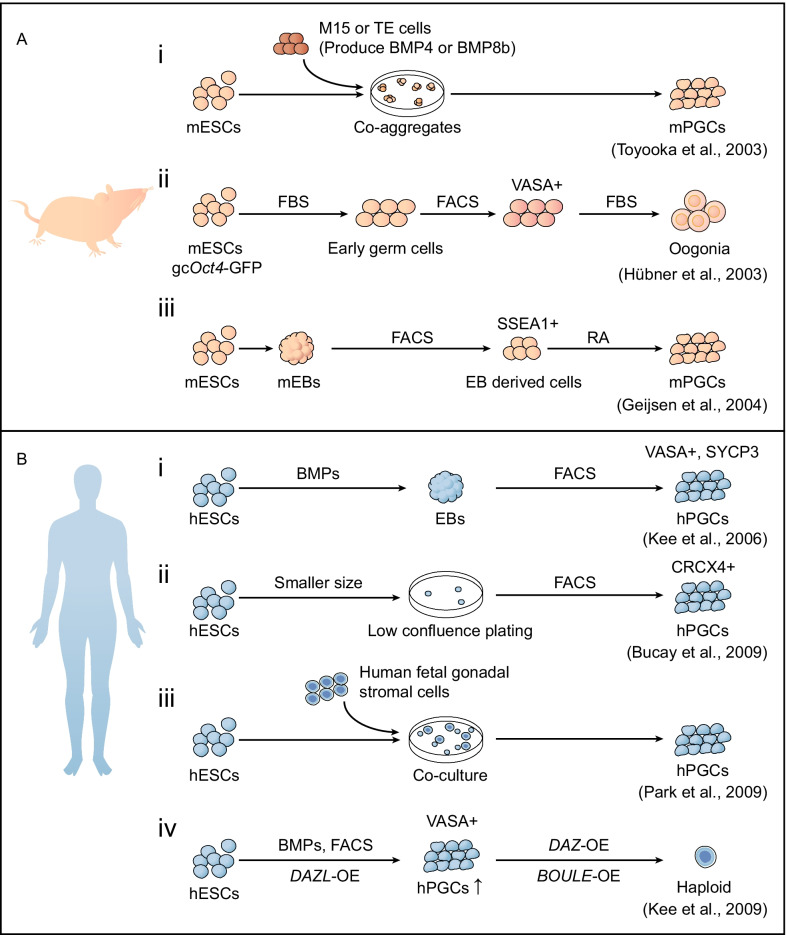
Traditional induction of germ cells from ESCs in the 2000s. **A** Schematic of mouse germ cell specification in the 2000s. (i) mESCs were cocultured with M15 cells or trophoblast cells that secreted BMP4 or BMP8b to induce the specification of mPGCs [22]. (ii) gc*Oct4*-GFP mESCs were cultured for 7 days in the presence of FBS to induce early germ cells, from which the VASA+ cells were enriched by fluorescence-activated cell sorting (FACS) and further cultured to generate oogonia [23]. gc*Oct4*-GFP,conserved region 2 (CR2) and CR3, also termed the proximal enhancer, were deleted from the 5’ regulatory region of *Oct4*. OCT4 and GFP were driven by the distal enhancer. FBS, fetal bovine serum,VASA, a marker of postmigratory germ cells. (iii) EBs were formed from ESCs, and EB-derived SSEA1^+^ cells were enriched by FACS and further cultured with RA to induce mPGCs [24]. RA, retinoic acid. **B** Schematic of human germ cell specification in the 2000s. (i) hESCs were aggregated into hEBs when treated with BMPs, from which VASA+ and SYCP3+ double-positive cells with PGC identities were enriched by FACS [26]. (ii) hPGCs can be produced by reducing the size and confluence of plated hESC colonies. These cells could be purified by sorting with CXCR4 antibody [27]. (iii) hESCs were cocultured with human fetal gonad stromal cells to facilitate the production of hPGCs [28]. (iv) Overexpression of DAZL could promote the efficiency of hPGC (VASA+ cells sorted by FACS) specialization from hESCs with BMPs. In addition, co-overexpression of DAZ and BOULE could promote the meiosis of PGCs [29].

In 2003, Yayoi et al. co-aggregated mESCs (carrying a mouse *Vasa* homolog (MVH) reporter) with trophoblast cells or M15 cells (producing BMP4 or BMP8b) and obtained 2.9% MVH-positive cells from the aggregates within a day. These MVH-positive cells could participate in spermatogenesis when transferred into testicular tubules [22]. In the same year, Hübner et al. proved that mESCs could enter meiosis to produce oogonia. They enriched PGCs with gc*Oct4*-GFP and *Vasa* double positivity and further cultured them with FBS to form oogonia [23]. To produce male gametes in vitro, Niels et al. cultured EB-derived SSEA1-positive cells in the presence of retinoic acid (RA) to induce mPGCs and haploid gametes from mESCs [24]. Thereafter, a more comprehensive study revealed that retinoid signaling (role of RA) determined the cell fate of germ cells in mice [25]. Early studies of germline specification focused mainly on the generation of PGCs, however, whether functional haploid gametes could be obtained by differentiation needs more investigation (Fig. 1A).

Human germline development is rarely studied due to the shortage and inaccessibility of human embryos for ethical reasons. As hESCs and mESCs are genetically and epigenetically different [30], strategies for germline specification of hESCs might be different from those for mESCs (Fig. 1B). In 2004, hESCs were differentiated randomly in aggregates, expressing some germ cell-specific markers, including *VASA*, *BOL*, and *SCP1* [31]. Next, Kee et al. found that the differentiation efficiency of PGCs can be improved with BMPs by enrichment for VASA and SYCP3 double-positive cells [26]. Bucay et al. reduced the size of cultured ESC colonies with low confluence to plates and obtained hPGCs by enrichment for CXCR4-positive cells [27]. In addition, Park et al. found that coculturing hESCs with human fetal gonadal stromal cells significantly improved the generation efficiency of hPGCs [28]. Another report revealed that overexpression of *DAZL* promotes germ cell differentiation from hESCs, further yielding haploid cells through the overexpression of *DAZ* and *BOULE* [29]. Although germ cells expressing specific markers were obtained from mESCs and hESCs, whether authentic PGCs with the potential to function as gametes could be generated from PSCs is still unclear and of great interest.

## Fertile gametes from pluripotent stem cells

**Fig. 2.**
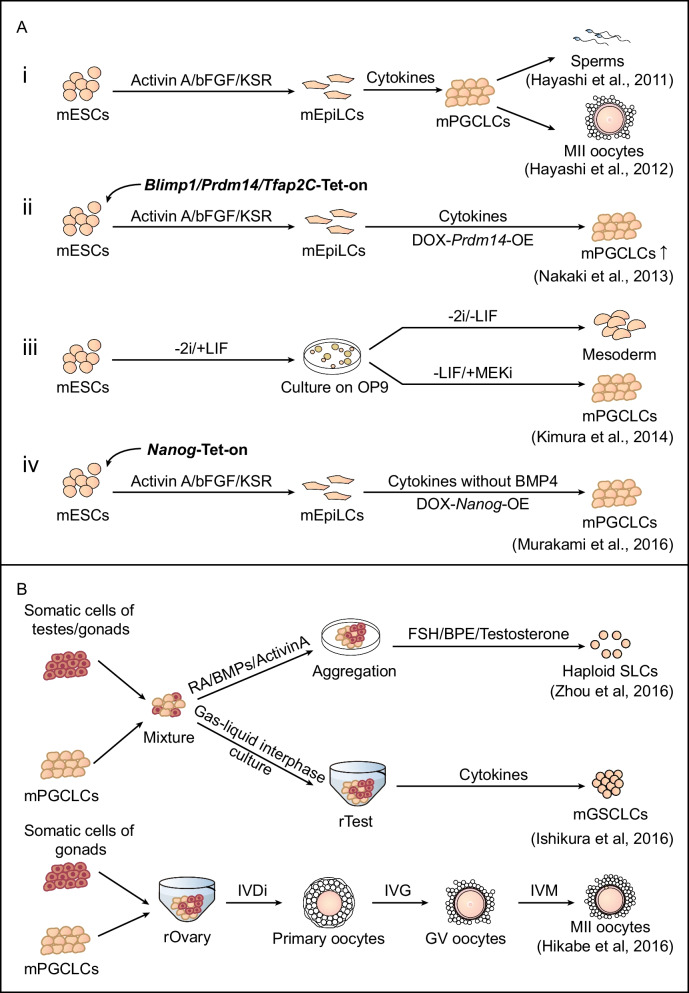
Generation of fertile gametes from ESCs in mouse. **A** Schematic of mouse germline specification in vitro. (i) mESCs were induced to differentiate into EpiLCs for 2 days in the presence of Activin A and bFGF. mEpiLCs were induced into mPGCLCs for 6 days by cytokines including BMP4 and BMP8. mPGCLCs were able to form fertile spermatozoa or oocytes in vivo [5, 6]. (ii) mESCs were transfected with DOX-inducible vectors, differentiated into mPGCLCs through a similar EpiLC process, and then aggregated by adding DOX to induce overexpression of *Blimp1, Prdm14* and *Tfap2C*. The efficiency of PGC derivation was improved just by overexpression of *Prdm14* [35]. (iii) mESCs were cultured without 2i before induction for 3 days and cocultured with OP9 cells with MEK inhibitor and without LIF to induce the PGC fate [38]. 2i, GSK inhibitor and MEK inhibitor. (iv) mESCs were induced to differentiate into EpiLCs as described above, which were further induced into mPGCLCs without cytokines by overexpression of *Nanog* [39]. **B** Schematic of gamete specification from mPGCLCs in vitro. (i) Male mPGCLCs were cocultured with testicular cells supplemented with RA, BMPs, Activin A and testosterone, FSH and BPE to form mSLCs [9]. Male mPGCLCs were aggregated with somatic cells from embryonic gonads to form reconstituted testes (rTestes) by gas-liquid interphase culture and then cultured with GNDP, bFGF, LIF and EGF to form mGSCLCs [8]. mSLCs, mouse spermatid-like cells,mGSCLCs, mouse germline stem cell-like cells. (ii) Female mPGCLCs were aggregated with E12.5 gonadal somatic cells to form reconstituted ovaries (rOvaries). rOvaries were cultured and differentiated for 5 weeks to further produce mature oocytes [7].

Although there have been many studies of PGC specification from ESCs in mice, their further differentiation to fertile gametes is still challenging. In mice, germ cell fate is initialized at the epiblast beginning at E5.75, which can be indicated conveniently with *Blimp*-mVenus (BV) and *Stella*-ECFP (SC) reporters [32]. Hayashi et al. cultured mESCs in a “2i/LIF” condition [33] and differentiated them in the presence of activin A and bFGF to induce epiblast-like cells (EpiLCs), which exhibited similarities to the post-implantation epiblast at E5.75 [6]. These EpiLCs were further aggregated with defined cytokines, including BMP4 [34] and other factors, for several days (Fig. 2A). Typical PGC-like cells (PGCLCs) could be enriched from aggregates with BVSC reporters or SSEA-1 and CD61 antibodies. These PGCLCs were transferred back into testes or ovaries, and both could further produce reproductive gametes [5, 6]. Through overexpression of *Prdm14*, EpiLCs can also swiftly and efficiently differentiate into PGCs [35]. It is known that coculture of mESCs with OP9 cells induces mesodermal differentiation in vitro [36, 37]. Using these mesodermal differentiation protocols, PGCLCs were efficiently induced from ESCs by coculture with OP9 cells and inhibition of ERK signaling, attributed to the upregulation of germ cell marker genes and downregulation of mesodermal genes [38]. The overexpression of *Nanog* in mEpiLCs could induce mPGCLCs, independent of BMP4. In addition, NANOG could bind to and activate the enhancers of *Blimp1* and *Prdm14* [39]. Altogether, this “EpiLC-based aggregates with defined cytokines” method greatly promoted the derivation of PGCLCs, while more specific double-positive enrichment and clearer germ cell regulation pathways guaranteed the authentic identities of derived PGCLCs.

Given that in vitro-derived PGCLCs could generate functional gametes when transferred back to germlines in vivo, it was fascinating to address whether PGCLCs were able to produce functional gametes in vitro. In 2016, Zhou et al. cocultured SSEA1 and CD61 double-positive PGCLCs with testicular cells in the presence of defined cytokines and hormones. Haploid spermatid-like cells (SLCs) were generated from the cell cultures and were capable of producing offspring *via* intracytoplasmic injection [9]. Another group cocultured PGCLCs from ESCs with gonad-derived somatic cells in a gas-liquid interphase culture system, which further generated propagating spermatogonial stem cells [8]. In the same year, fertile oocytes were also obtained from cocultures of ESC-derived PGCLCs and gonad-derived somatic cells [7]. Thereafter, Ohta et al. developed a chemically defined culture system in which PGCLCs could expand 50-fold while still maintaining robust capacity for spermatogenesis [40]. Besides in the presence of forskolin and rolipram, cyclosporin A (CsA) and fibroblast growth factors (FGFs: FGF2 and FGF10) effectively enhanced nearly 50-fold during the expansion of mPGCLCs in vitro. And mPGCLCs which expanded exposed in CsA and FGFs comprehensively erased their DNA methylation like the wild type gonadal germ cells in vivo [41]. Based on this culture system, Ishikura et al. reconstituted whole male germ-cell development from mESCs in vitro [42]. Thus, both male and female gametes could be produced by in vitro mESCs using various strategies (Fig. 2B), which is beneficial for basic studies of germline development and clinical demands.

**Fig. 3.**
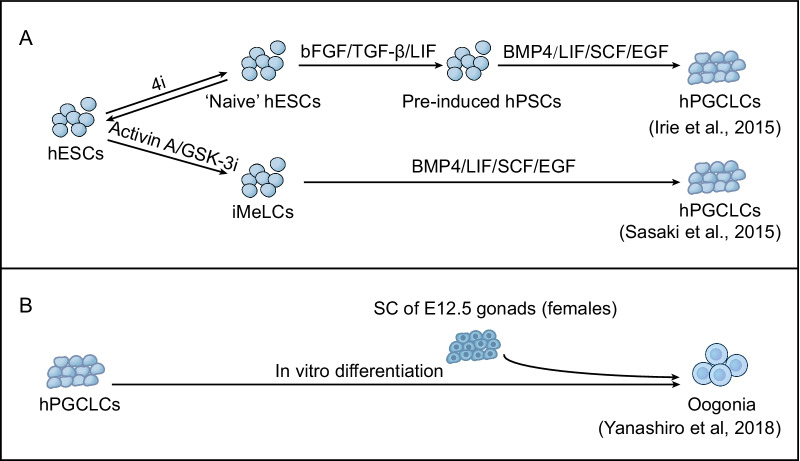
Generation of hPGCLCs from ESCs in 2010s. **A** Schematic of hPGCLC specification in vitro. “Naive” hESCs were generated by treatment of hESCs with 4i for 3-5 days first, and then the hPSCs were preinduced when exposed to cytokines to further produce hPGCLCs [21] (top). hPSCs were induced to differentiate into iMeLCs in the presence of activin A and GSKi for 2 days. Then, iMeLCs were induced into hPGCLCs by cytokines, including BMP4, for 8 days [44] (bottom). 4i, ERKi, GSKi, JNKi, p38i,iMeLCs, incipient mesoderm-like cells. **B** Schematic of gamete specification from hPGCLCs in vitro. hPGCLCs cocultured with somatic cells from E12.5 embryonic gonads could further differentiate into oogonia precursor states [45].

Reconstitution of the mouse germline in vitro is well developed, providing a platform for infertility research. The successful specification of germline cells from ESCs is mainly attributable to two factors: (1) initiation from the EpiLC state and (2) induction conditions including BMP signals. In addition, the derivation efficiency of mPGCLCs could be improved through the overexpression of transcription factors including *Prdm14* and *Nanog*. Since the pluripotent state of hESCs was totally distinct from that of mESCs, the specification of PGCs from hESCs required different methods (Fig. 3A). Irie et al. cultured hESCs in ‘‘4i’’ medium [43] to induce a ground state resembling mESCs. Thereafter, “4i hESCs” were cultured in the presence of bFGF, TGFβ and LIF to obtain “preinduced hESCs”, similar to mEpiLCs. Finally, with cytokines, including BMP4, “preinduced hESCs” differentiate into hPGCLCs [21]. Another group induced hiPSCs into primary mesoderm-like cells (iMeLCs) by adding Activin A and CHIR99021, which can stably produce hPGCLCs. The iMeLCs were cultured in the presence of BMP4, SCF, EGF and LIF and differentiated into *Blimp1*-tdTomato and *AP2γ*-EGFP double-positive hPGCLCs expressing PGC-specific genes, including *Blimp1* [44]. To explore the differentiation potential of hPGCLCs, Yamashiro adopted a xenogeneic ovary strategy to aggregate hPGCLCs with mouse embryonic ovary somatic cells. After approximately 10 weeks of culture, hPGCLCs underwent epigenetic reprogramming and differentiated into oogonia structures (Fig. 3B). In another 4 months of culture, the cell cultures began to express some key genes of premeiotic cells [45].

## Key Regulators of PGC Specification

**Fig. 4.**
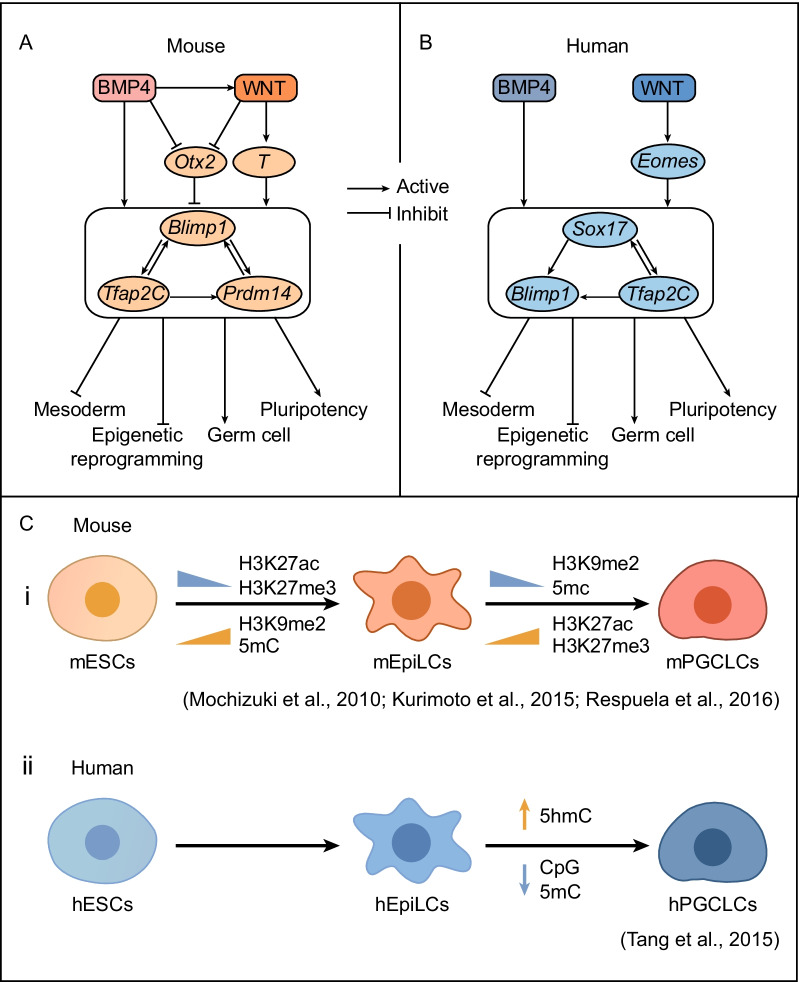
Regulatory pathways and epigenetic modifications related to germline specification. **A** Regulatory pathways for PGC specification in mice. The BMP4 signaling pathway directly or indirectly activates the WNT signaling pathway, which activates T cells. T cells directly activate *Blimp1* and *Prdm14*. *Tcfap2c* is a downstream target of *Blimp1*, enabling PGCs to maintain *Blimp1* activity. *Prdm14* activates *Tfap2C,* which enhances the activity of *Blimp1*, while the expression of *Blimp1* activates the expression of *Tfap2C*. *Otx2* functions repressively upstream of PGC transcription factors and is inhibited by the BMP4 and WNT signaling pathways. **B** Regulatory pathways for PGC specification in humans. The mechanism underlying hPGC specification was distinct from that of mPGC specification. *Sox17* plays an important role in hPGC specification. The BMP4 signaling pathway initially activates *Sox17*, whereas WNT activates *Eomes*, which further activates *Sox17. Sox17* is upstream of *Blimp1* in the PGC specification circuit. *Blimp1* can be upregulated by *Tfap2C*, which is upregulated by *Sox17* in response to the BMP4 signal. **C** Epigenetic modifications during the induction from ESCs to PGCLCs in mice and humans

PGC specification consists of three key events: repression of somatic programming, regaining of pluripotency and genome-wide epigenetic reprogramming. The transcription factors (TFs) *Blimp1* (also known as *Prdm1*) and *Prdm14* play important roles in mPGC formation [14, 16]. Prdm1 deficiency might cause PGC apoptosis [46]. *Prdm14* is a PR domain-containing transcriptional regulator that regulates pluripotency and genome-wide epigenetic reprogramming and is specifically expressed in the germline and PSCs. In *Prdm14*-deficient embryos, cells destined to become PGCs were unable to regain pluripotency and fulfil epigenetic reprogramming [16, 47]. Functionally, *Blimp1* inhibited the differentiation of EpiLCs to mesoderm lineages, whereas *Prdm14* inhibited neural differentiation and activated *Blimp1*. The TF *Tfap2c* was a downstream target gene of *Blimp1*, which was specifically expressed in E7.25-E12.5 PGCs population [48]. In addition, *Sall4* is involved in PGC specification in conjunction with *Blimp1* to recruit repressor complexes of somatic cell fates [49]. The co-overexpression of *Blimp1*, *Prdm14* and *Tfap2c* enabled EpiLCs to differentiate into PGCLCs efficiently, indicating their core roles in the induction of PGCLCs [35]. In mice, it had been proven previously that cells respond to BMP4 by expressing *Blimp1*, *Prdm14* and *Tfap2c*. The wingless/INT-1 (WNT) pathway responds to BMP-induced signals to control PGC specification [34]. *T* (also known as *Brachyury*) was subsequently found to have a similar function, and the genetic disruption of *T* prevented the expression of early mPGC markers as *Prdm1* and others [50]. Recently, Zhang et al. found *Otx2* to be a key roadblock to the differentiation of germline fate. During the specification of PGCs, BMP4 and the activated WNT pathway repressed the expression of *Otx2*. When OTX2 is absent, the induction of PGCLCs does not require BMP4 or BLIMP1 [51]. Pluripotent TFs also play important roles in the development and survival of PGCs. For example, *Oct4* is a heterodimeric mate of *Sox2*, which is also required for PGC specification [52]. *Oct4* is continuously expressed in epidermal cells due to its role in PGC fate commitment, but the expression of *Nanog* and *Sox2* is downregulated in epidermal cells in the pregerm stage and re-expressed in PGCs [53, 54]. The results of single-cell analysis of early mPGCs were consistent with these findings [55]. All of the above are the regulatory pathways involved in mouse germline specification (Fig. 4A).

Similar to the case in mPGCs, conserved genes such as *BLIMP1*, *TFAP2C*, *NANOS3* and *NANOG* are also active in humans. However, the regulatory pathways are different from those in mice (Fig. 4B). The core regulators of PGC specification in humans are *BLIMP1*, *TFAP2C* and *SOX17* but not *PRDM14*. The disruption of *SOX17* caused the failure of hPGC specification, while the overexpression of *SOX17* was able to improve the efficiency of hPGC specification [21]. In addition, *GATA3* or *GATA2* as the immediate BMP effectors, combined with *SOX17* and *TFAP2C* was able to promote hPGCLCs generation [56]. *EOMES* encodes many T-box-containing TFs and is required for the development of extraembryonic ectoderm in mouse post-implantation embryos and for gastrulation [57]. In humans, *EOMES* is upstream of *SOX17* and downstream of the WNT pathway, which activates the expression of *SOX17* and induces the specification of hPGCs [58]. In addition, *SOX17* could activate *BLIMP1*, further upregulating *TFAP2C* [48]. Interestingly, the expression of *TFAP2C* was independent of *SOX17* but responded to BMP4 [58]. Recently, scientists analyzed the landscape of hPGC specification and found that the differentiation from PSCs to germ cells was driven by the core TF network of *OCT4*, *SOX2*, *PAX5* and *PRDM1* [59]. The regulatory network of hPGC specification from PSCs was identified gradually through CPISPR/Cas9-based genetic screening [60].

In PGC specification, in addition to TF regulation, epigenetic reprogramming was essential to erase parental imprinting (Fig. 4C). Correct epigenetic reprogramming leads to the normal development of offspring. Mouse PGCs underwent the demethylation of genomic DNA, erasure of imprinting, upregulation of H3K27me3, and downregulation of H3K9me2 in vivo [61, 62]. H3K9me2 levels were inhibited at E7.25, and H3K27me3 levels increased at E8.25 [63]. In mice, the major histone modifications in PGCLC specification include H3K27me3, H3K4me3 and H3K9me3. ESCs differentiate into EpiLCs with the change in H3K27 acetylation (H3K27ac) that occurs at this stage [64]. With the enrichment of H3K27ac, T activates the mesoderm program and the expression of *Blimp1* [65]. H3K27ac is then decommissioned and becomes H3K9me2 in EpiLCs [64]. Bivalency of H3K4me3 and H3K27me3 marks poised promoters [20]. The level of H3K27me3 decreased through the activation of the mesoderm program, while during the induction of PGCLCs, H3K27me3 reappeared, and BLIMP1 acted as a potential nucleator for the enrichment and spreading of H3K27me3 [65]. Meanwhile, methylation was regulated by the expression of *Prdm14*. The level of 5mC increased during the transition from mESCs to EpiLCs but decreased during the induction from EpiLCs to PGCLCs [66, 67]. DNA methylation reprogramming in human PGC specification was similar to that in mice. By the expression of *SOX17* and *BLIMP1*, *de novo* DNA methylation was repressed in hPGCLCs, and 5hmC was globally enriched, while the level of 5mC decreased [68]. The CpG levels in hiPSCs and iMeLCs were high initially and then decreased during the induction of hPGCLCs [44, 69].

## Conclusions

PGCLCs have been achieved from PSCs in mice and humans and are promising for the reconstitution of the germline in vitro. Furthermore, although mPGCLCs can be successfully induced into functional oocytes and spermatozoa, the efficiency of spermiogenesis in vitro is still low, indicating that more mechanisms underlying spermiogenesis need to be clarified. All the findings related to germline specification in mice have facilitated the studies of hPGCLC generation; nevertheless, how hPGCLCs can be induced into haploid gametes warrants further investigation. Since the regulatory pathways involved in human germline specification are distinct from those in mice, it is necessary to focus on the core module controlling this process, similar to the role of *Otx2* in mice.

## Data Availability

Not applicable.

## Abbreviations

ESCs: Embryonic stem cells
PSCs: Pluripotent stem cells
PGCs: Primordial germ cells
PGCLCs: PGC-like cells
iPSCs: Induced pluripotent stem cells
mPGCs: Mouse PGCs
E5.25–6.25: Embryonic day 5.25–6.25
AP: Alkaline phosphatase
hPGCs: Human PGCs
RA: Retinoic acid
BV: *Blimp*-mVenus
SC: *Stella*-ECFP
EpiLCs: Epiblast-like cells
SLCs: Spermatid-like cells
iMeLCs: Induced primary mesoderm-like cells
WNT: Wingless/INT-1
H3K27ac: H3K27 acetylation
